# Integrative Transcriptomics and Mendelian Randomization Identify *RGS1* as a Causal Immune Regulator in Alzheimer’s Disease

**DOI:** 10.3390/cimb48080828

**Published:** 2026-08-14

**Authors:** Zhiyun Cheng, Ruyu Bai, Yong Diao

**Affiliations:** School of Medicine, Huaqiao University, Quanzhou 362021, China; chengzhiyun@hqu.edu.cn (Z.C.); bairuyu@hqu.edu.cn (R.B.)

**Keywords:** Alzheimer’s disease, Mendelian randomization, *RGS1*, neuroinflammation, immune microenvironment

## Abstract

Alzheimer’s disease (AD) has a complex pathogenesis, involving molecular and neuroimmune dysregulation, but the causal drivers linking transcriptomic changes to immune remodeling are not yet clear. In a discovery cohort, differential expression analysis was performed, and it was independently validated in two external cohorts. Key genes were prioritized via LASSO/logistic regression, functionally annotated, and causally linked to AD using two-sample MR. The neuroimmune landscape was mapped by ssGSEA, and top candidates were validated in vitro. *RGS1* is a key node in neuroinflammation and cytoskeletal dynamics, which is prioritized by the algorithmic intersection. MR analysis suggested a potential causal association between genetically predicted *RGS1* expression and AD risk. *RGS1* was consistently upregulated in both discovery and validation cohorts (AUC: 0.61–0.67) and was confirmed in vitro. Immune deconvolution showed that AD-specific enrichment profiles occur, and *RGS1* is strongly correlated with activated CD4+ T cells and pro-inflammatory chemokines. *RGS1* is identified as a robust key gene that may contribute to immune microenvironment dysregulation in AD, and combining discovery-validation transcriptomics, causal inference, and experimental validation, we find that *RGS1* is a potential immunomodulatory target.

## 1. Introduction

Alzheimer’s disease (AD) is a progressive neurodegenerative disorder characterized by amyloid-β accumulation, tau pathology, and multifactorial cellular dysfunction [1]. Although a large number of studies have been conducted, therapeutic strategies that can change the pathogenesis of the disease have not been realized yet because the intricate cellular environment does not separate initial genetic risks from severe clinical deterioration [2]. Traditional transcriptomics of post-mortem brain tissue have found that neurotransmission, calcium homeostasis, and energy metabolism are all significantly affected [3]. These transcriptomic changes often represent secondary compensatory mechanisms or pathological consequences rather than primary initiating events, making them suboptimal therapeutic targets [4].

High-dimensional RNA-seq data are inherently multicollinear and susceptible to technical batch effects, rendering conventional univariate filtering prone to poor cross-cohort reproducibility [5]. The gene signatures of the conventional univariate filters are different in the validation sets [6]. Penalized regression approaches, such as LASSO (L1-regularization) combined with multivariate logistic modeling, enable sparse feature selection and improve biological interpretability [7]. Furthermore, conventional observational correlations do not equate to causal relationships [8]. The two-sample Mendelian randomization based on cis-eQTLs can be used to study the causal effect of altered mRNA expression on disease risk without the problem of confounding bias or reverse causation in epidemiological genetics [9].

Neuroinflammation is one of the characteristics of AD, and genetic and neuropathological research has been conducted [10]. Genome-wide association studies have often found that microgliosis, the complement pathway, and extracellular immune factors are related to AD [11]. Single-cell analysis and spatial mapping have shown that in the development of AD, there is significant remodeling of the cerebral innate microenvironment, irregular migration of T cells, increased dendritic cell infiltration, and enhanced checkpoint signaling [12]. However, the transcriptional intermediates connecting genetic variants to immune activation or cell states are still not well known [13]. Considering that the clinical features of AD are varied and subtle, more attention has been paid to the overall function of regulatory elements in all contexts rather than to individual single-site mutations [14]. If the prior assumption is not biased, then a computational pipeline that uses only empirical data can make full use of the rich resources of transcriptomic databases and the statistical power of dimension reduction methods and genetic causality knowledge to find the root cause of the disease, instead of secondary inflammation leading to neurodegeneration [15]. In addition, with the help of in silico prediction tools and spatially detailed immunology maps, as well as recursive experimental trials, it has been shown that observational correlations can be converted into verifiable physiological mechanisms [16,17]. In summary, this study aims to systematically identify key genes regulating the neuroimmune environment in AD, and propose genetically supported mechanisms for novel immunotherapeutic development.

To address these knowledge gaps, we developed an integrated analytical pipeline combining discovery-validation transcriptomics, penalized feature selection, cis-eQTL-based MR, and in vitro validation. Differential expression profiling was performed in a discovery cohort and independently replicated across two external GEO datasets. Consensus key genes were identified through multivariate logistic regression and LASSO coefficient shrinkage, followed by functional enrichment and causal inference via two-sample MR. Cross-cohort reliability was further substantiated by protein-level verification in an okadaic acid-induced AD-like cellular model. This study aims to identify and causally validate key regulatory genes orchestrating neuroimmune dysregulation in AD, with a focus on *RGS1* as a potential immunomodulatory target for disease-modifying intervention.

## 2. Materials and Methods

### 2.1. Data Acquisition

**Figure 1:**
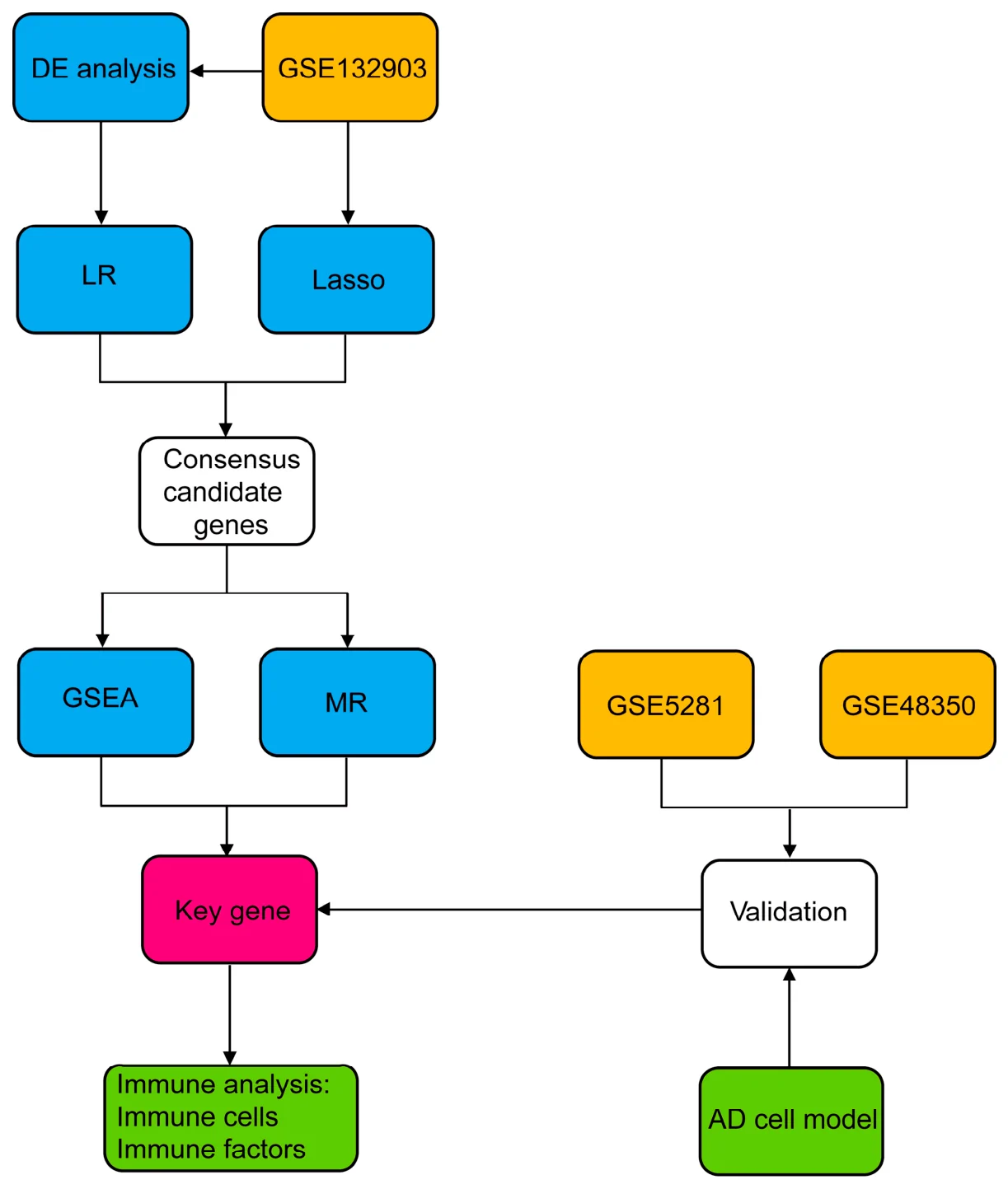
General diagram of the entire analytical framework. Color code: Orange represents the discovery and identification of collections; blue indicates transcriptional data processing and comparative analysis; green is for digital models coupled with quantitative consolidation; pink highlights the identified essential key genes subjected to further predictive and functional analyses.

The Gene Expression Omnibus (GEO) database (https://www.ncbi.nlm.nih.gov/geo, accessed on 18 November 2025) was used to obtain the expression arrays. Three independent cohorts with well-characterized clinical annotations were included: GSE132903 has 97 confirmed AD patients and 98 non-demented (ND) individuals, and it was derived from the middle temporal gyrus; GSE48350 has matched 80 AD subjects with 173 ND cases, and it originated from the hippocampus, entorhinal cortex, superior frontal cortex and postcentral gyrus; and GSE5281 has merged 74 AD samples with 87 ND samples, and the brain areas covered include the entorhinal cortex, hippocampus, medial temporal gyrus, posterior cingulate, superior frontal gyrus and primary visual cortex. Piras et al. [18], Berchtold et al. [19] and Liang et al. [20] conducted the first investigations on these assemblies separately. The detailed demographic and clinical characteristics of all datasets, including the distinct counts of subjects and tissue samples, are summarized in Supplementary Table S1. The whole calculation pipeline is shown in Figure 1.

### 2.2. Differential Expression Analysis

The GSE132903 dataset served as the discovery cohort, and then the R package limma (version 3.40.6) was employed to carry out differential gene expression analysis of AD patients and ND controls. A linear model was fitted via lmFit, followed by empirical Bayes moderation of standard errors with eBayes to compute moderated t-statistics and log_2_ fold changes (log_2_FC). Genes with large changes were selected based on the criteria of |log_2_FC| > 0.585 and adjusted *p* < 0.05.

### 2.3. Functional Enrichment Analysis

ClusterProfiler R tool (version 3.14.3) was employed to carry out categorical functional analysis of the Gene Ontology (GO) database and the Kyoto Encyclopedia of Genes and Genomes (KEGG) database. Currently, the KEGG pathway data are obtained by calling the KEGG REST API, and the GO information from org.Hs.eg.db (version 3.1.0) is used as the main database system. We have set a lower bound of 5 for the number of genes and an upper bound of 5000 in this study. Only the KEGG pathways with *p* < 0.05 and FDR < 0.05 were taken to be statistically significant.

### 2.4. Multivariate Logistic Regression

Multivariate logistic regression is employed here to study the collective predictive effects of all the DEGs in AD. Transcriptomic expression values were Z-score normalized prior to modeling. A fixed stochastic seed was used to divide the whole microarray dataset into training (80%) and validation (20%) sets. To address potential multicollinearity among predictors, the variance inflation factor (VIF) was calculated, and attributes with a VIF greater than 5 were gradually excluded. Maximum likelihood estimation was used to construct the polished model. Five-fold cross-validation was carried out on the training set to ensure that there was no overfitting. The remaining validation set was used to evaluate how well the scheme could make predictions, and then AUC-ROC, accuracy, sensitivity, negative predictive value, F1-score, etc., were used to quantify it. Calibration plots were generated, and the Hosmer–Lemeshow goodness-of-fit test was performed on the validation set. All modeling procedures were implemented in R using the caret, stats, pROC, and rms packages. A two-tailed *p* < 0.05 was taken as the sign of statistical significance. The construction and display of the models were in line with the TRIPOD rules.

### 2.5. Lasso Regression

Lasso regression was used to perform feature selection optimization, and it was carried out using the glmnet package in R. A 10-fold cross-validation was used to select the best value of the penalty parameter (λ). λ was selected according to the value minimizing binomial deviance (lambda.min) to prioritize model parsimony, yielding a final λ value of 0.06.

### 2.6. Venn Diagram Analysis

To identify robust candidate genes, the feature sets derived from LASSO-penalized regression and multivariate logistic regression were intersected. Genes retained in both screening pipelines were defined as consensus predictors for downstream validation. Set intersections were visualized using the R package VennDiagram (v1.7.3).

### 2.7. Gene Set Enrichment Analysis (GSEA)

To determine the different expression patterns of *RGS1*, *RNVU1-7,* and *VGF* systematically, GSEA (version 3.0) was used. All samples were stratified into high- and low-expression subgroups based on the median transcript levels of each target gene. c2.cp.kegg.v7.4.symbols.gmt biological database was downloaded from the MSigDB official website. The number of phenotype permutations for enrichment evaluation is 1000, and the minimum gene set threshold is 5; however, each category may have as many as 5000 components. Pathways were considered significantly enriched at a nominal *p* < 0.05 and FDR < 0.25, consistent with standard GSEA reporting guidelines established by the Broad Institute.

### 2.8. Mendelian Randomization Analysis

To evaluate the potential causal effect of genetically predicted *RGS1* expression on AD risk, a two-sample Mendelian randomization (MR) analysis was conducted. Information on quantitative trait loci (cis-eQTL) for the cis-expression of *RGS1* was obtained from the IEU OpenGWAS repository (dataset ID: eqtl-a-ENSG00000090104). SNPs within ±500 kb of the *RGS1* transcriptional start site were selected as the instrumental variables (IVs), and all of them had a *p*-value of less than 5 × 10^−6^ (a relaxed threshold commonly applied in cis-eQTL MR to ensure sufficient instrument strength). Pruning of linkage disequilibrium was carried out according to the 1000 Genomes European panel (r^2^ < 0.01 in a 5000 kb interval), and only those SNPs that met this condition were selected. Weak instrument bias was assessed via the F-statistic, with all retained IVs exceeding the conventional threshold of F ≥ 10. The summary statistics of AD were obtained from the European ancestry subset of IEU OpenGWAS (ieu-b-5067), which had 954 cases and 487,331 controls. Exposure and outcome datasets were harmonized by aligning effect alleles, and palindromic SNPs with a minor allele frequency (MAF) > 0.42 were excluded to resolve strand ambiguity. The main causal estimate is obtained by means of the inverse-variance weighted (IVW) method in a multiplicative random-effects model. All of the analysis work was done with the TwoSampleMR (version 0.5.11) and MRPRESSO (version 1.0) tools in R. Statistical significance was defined as a two-sided *p* < 0.05. This study adhered to the STROBE-MR reporting guidelines.

### 2.9. Receiver Operating Characteristic (ROC) Curve Analysis

The discriminative ability of *RGS1* expression in distinguishing AD cases from cognitively normal controls was evaluated using receiver operating characteristic (ROC) curve analysis. ROC curves were generated, and the area under the curve (AUC) was calculated using the R package pROC (version 1.17.0.1). In order to maintain the numerical stability of the AUC value and its corresponding 95% confidence interval, the ci tool in this computational environment was used to obtain them.

### 2.10. Western Blotting

Human neuroblastoma SH-SY5Y cells (catalog number CL-0208, Procell, Wuhan, China) were grown in DMEM/F12 medium (product code PM150312, Procell) with 10% fetal bovine serum (catalog number SH30084.03, Hyclone, Logan, UT, USA) at 37 °C in the laboratory according to the standard. Cells were divided into two groups: a control group and an AD model group, and both were in the proliferative stage. To create an AD-like state, 30 nM of OKA (catalog number HY-N6785, MCE, Shanghai, China) was added to the cells in the AD model group for 24 h, and at the same time, the control group was left in fresh medium without OKA. Lysis reagent (P013B, Beyotime, Shanghai, China) and 10% protease inhibitor cocktail (ST506, Beyotime) were added to extract the proteins from the cells. Bicinchoninic acid quantitative assay (catalog P0010, Beyotime) was used to determine the amount of protein after extraction, and then these lysates were thermally denatured. The same quantity of protein was used to carry out the SDS-PAGE separation and electrotransfer onto a PVDF membrane (catalog number IPVH00010, Millipore, Burlington, MA, USA), and then the results were obtained. Membranes were blocked with 5% non-fat milk in TBST buffer for 60 min at room temperature, followed by overnight incubation at 4 °C with primary antibodies (catalog number 12547-1-AP, Proteintech, Wuhan, China, 1:500; and Catalog 60004-1-Ig, Proteintech, 1:5000). After washing with TBST, membranes were incubated with secondary antibodies (catalog number 111-035-003 and 115-035-003, Jackson ImmunoResearch, West Grove, PA, USA; both 1:10,000). Immunoreactive bands were visualized using an enhanced chemiluminescence substrate (NCI5079, Thermo Fisher Scientific, Waltham, MA, USA) and quantified by densitometry using ImageJ software (Version 1.53k).

### 2.11. Immune Cell Enrichment Profiles and Analysis of Immune Factors

The relative abundance of 28 immune cell populations in the GSE132903 cohort was estimated using single-sample gene set enrichment analysis (ssGSEA), implemented via the R package IOBR (v0.99.9). Gene set signatures for immune cell subtypes were adopted from the curated 28-cell panel. To investigate the immunomodulatory role of *RGS1*, its transcript levels were correlated with a predefined panel of immune-related genes, comprising 20 inhibitory cytokines/checkpoints, 38 pro-inflammatory mediators, and 37 chemokine genes.

## 3. Results

### 3.1. Identification of Differentially Expressed Genes

**Figure 2:**
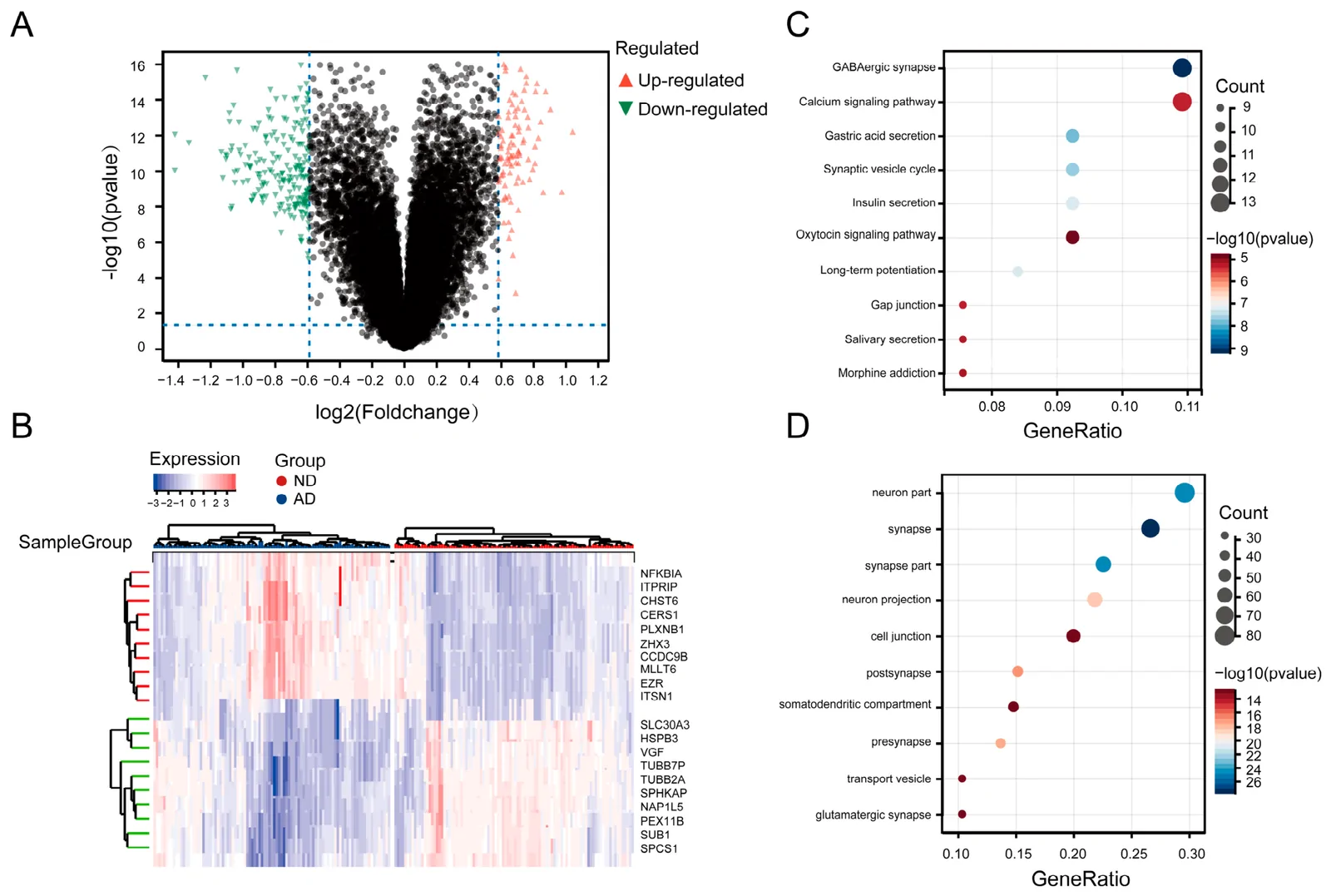
Transcriptomic alterations in AD. (**A**) Volcano plot summarizing differential gene expression between AD and ND groups, and the colors red and green indicate whether the genes are upregulated in the AD or ND groups, respectively, black represent non-differentially expressed genes. (**B**) Heatmap displaying the transcription profiles of the top 20 most significantly altered genes in AD patients compared to normal individuals. In the left dendrogram, the red branches represent a cluster of genes highly expressed in the AD group, whereas the green branches represent a cluster of genes highly expressed in the ND group. (**C**) The top five most significantly enriched KEGG pathways. (**D**) The top five most significantly enriched GO terms.

Transcriptomic profiling revealed distinct molecular signatures between AD brain tissues and ND cognitively normal controls. Comparative analysis revealed 302 differentially expressed genes (DEGs) that were found to be significantly altered and were then divided into 101 up-regulated genes and 201 down-regulated genes (Supplementary Table S2). A volcano chart is used to present the full allocation and quantitative importance of the changed expression values (Figure 2A). The top 20 significantly changed mRNAs, specifically the top 10 up-regulated and the bottom 10 down-regulated, are organized hierarchically and shown in a heatmap (Figure 2B).

### 3.2. Biological Functions and Pathway Enrichment of DEGs

To characterize the biological functions and pathways associated with the 302 identified DEGs, KEGG and Gene Ontology (GO) enrichment analyses were performed (Supplementary Table S3). According to the KEGG pathway classification of DEGs, there are 67 significant pathways (adjusted *p* < 0.05), predominantly involved in synaptic transmission and neuronal signaling. Key enriched terms included GABAergic synapse, long-term potentiation, calcium signaling pathway, and gap junction (Figure 2C). GO analysis identified 646 significantly enriched terms across biological processes (BP), cellular components (CC), and molecular functions (MF) (adjusted *p* < 0.05). The most significantly enriched CC terms were strongly associated with synaptic structures, including synapse, presynapse, postsynapse, and glutamatergic synapse. BP terms were primarily related to neuronal projection development, somatodendritic compartment organization, and vesicle-mediated transport. MF terms highlighted alterations in cell junction assembly and transporter activities (Figure 2D). Collectively, these enrichment profiles indicate a pronounced transcriptional dysregulation in synaptic architecture and neuronal signaling networks in AD brain tissues.

### 3.3. Multivariate Logistic Regression for 16 Genes in AD

**Figure 3:**
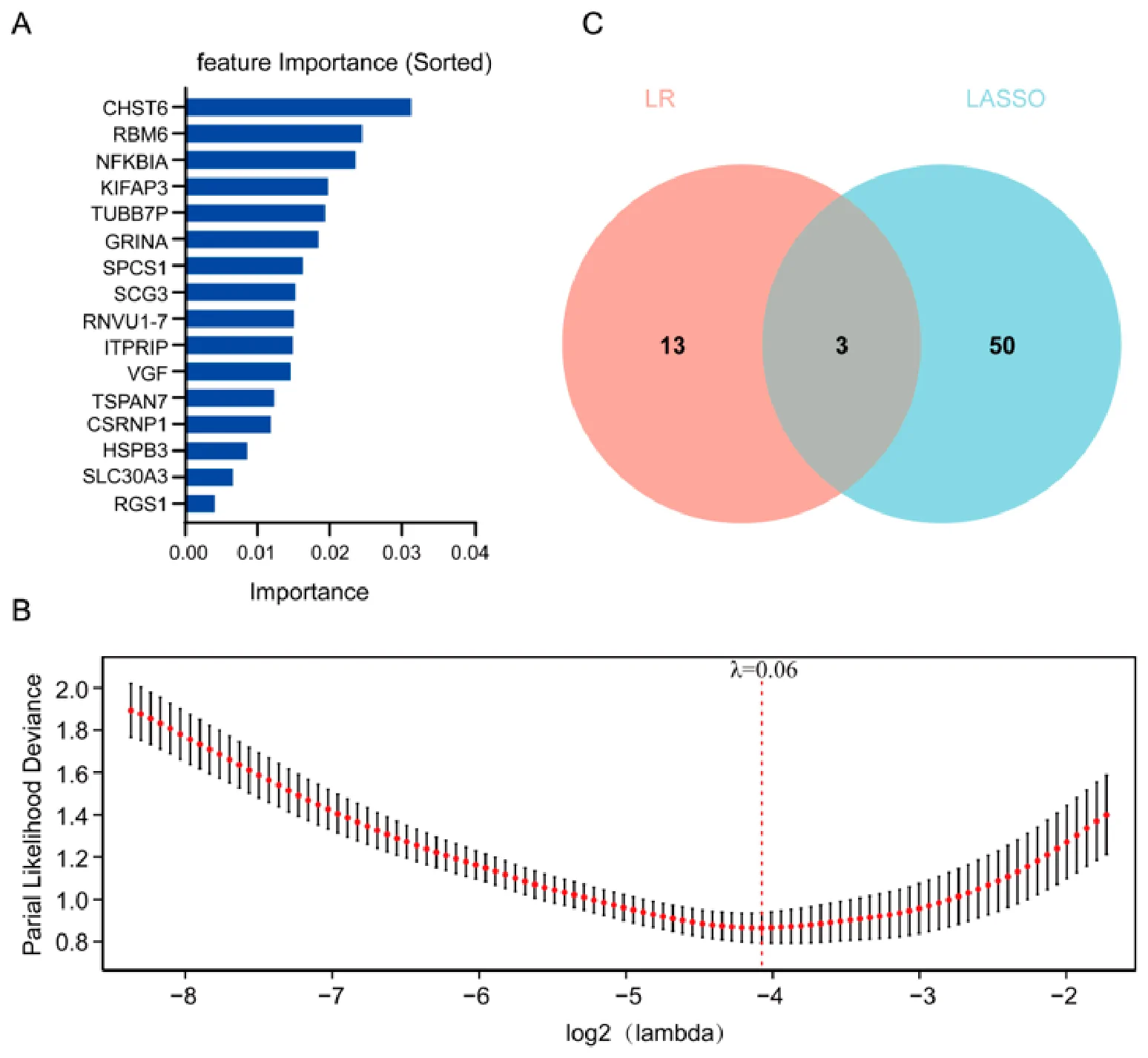
Identification of consensus candidate transcripts in AD via a dual-algorithm feature selection approach. (**A**) Multivariate logistic regression models were used to find patterns in clinical gene expression. (**B**) Feature dimensionality reduction was performed using LASSO-penalized regression. In the plot, the red dots represent the mean partial likelihood deviance, and the black vertical bars indicate the corresponding standard error (SE). The vertical red dashed line represents the optimal lambda (λ) value selected via cross-validation. (**C**) A Venn diagram is used to show the overlap of the required candidate genes from multivariate logistic regression and the non-zero coefficient variables obtained by LASSO regression. The overlapping area highlights the three core candidate genes selected for downstream causal inference and biological validation.

Based on the construction of a multivariate logistic regression model and feature selection, an experimental and characteristic molecular profile of all DEGs in AD vs. ND was obtained. Therefore, a set of 16 candidate genes was retained as independent predictors. The relative weight of a certain gene in the prediction was determined by normalized regression coefficients, and these values ranged from 0.0040 to 0.0313 (Figure 3A). *CHST6* had the highest effect at 0.0313, followed by *RBM6* (0.0245), *NFKBIA* (0.0236), and *KIFAP3* (0.0198). Additional genes with substantial predictive value included *TUBB7P* (0.0194), *ITPRIP* (0.0149), *VGF* (0.0145), *SPCS1* (0.0162), *SCG3* (0.0153), *GRINA* (0.0185), *TSPAN7* (0.0123), *CSRNP1* (0.0119), *RNVU1-7* (0.0151), *HSPB3* (0.0086), *SLC30A3* (0.0066), and *RGS1* (0.0040).

### 3.4. Feature Selection via LASSO Regression

To reduce dimensionality and mitigate overfitting among the initial DEGs, least absolute shrinkage and selection operator (LASSO) regression was applied. Ten-fold cross-validation was used to select an appropriate value for the regularization parameter (λ), and among all the penalty paths, the one that minimized binomial deviance was selected. At the selected λ value of 0.06, 53 genes retained non-zero coefficients and were retained as candidate predictors (Supplementary Table S4) (Figure 3B).

### 3.5. Identification of Consensus Discriminatory Genes via Intersection Analysis

To enhance the robustness of candidate genes and minimize algorithm-specific selection bias, we intersected the feature sets identified by multivariate logistic regression (16 genes) and LASSO regression (53 genes). Venn diagram analysis revealed an overlap of three genes: *RGS1*, *RNVU1-7*, and *VGF*, which were designated as the core signature for AD classification (Figure 3C).

### 3.6. Gene Set Enrichment Analysis (GSEA)

**Figure 4:**
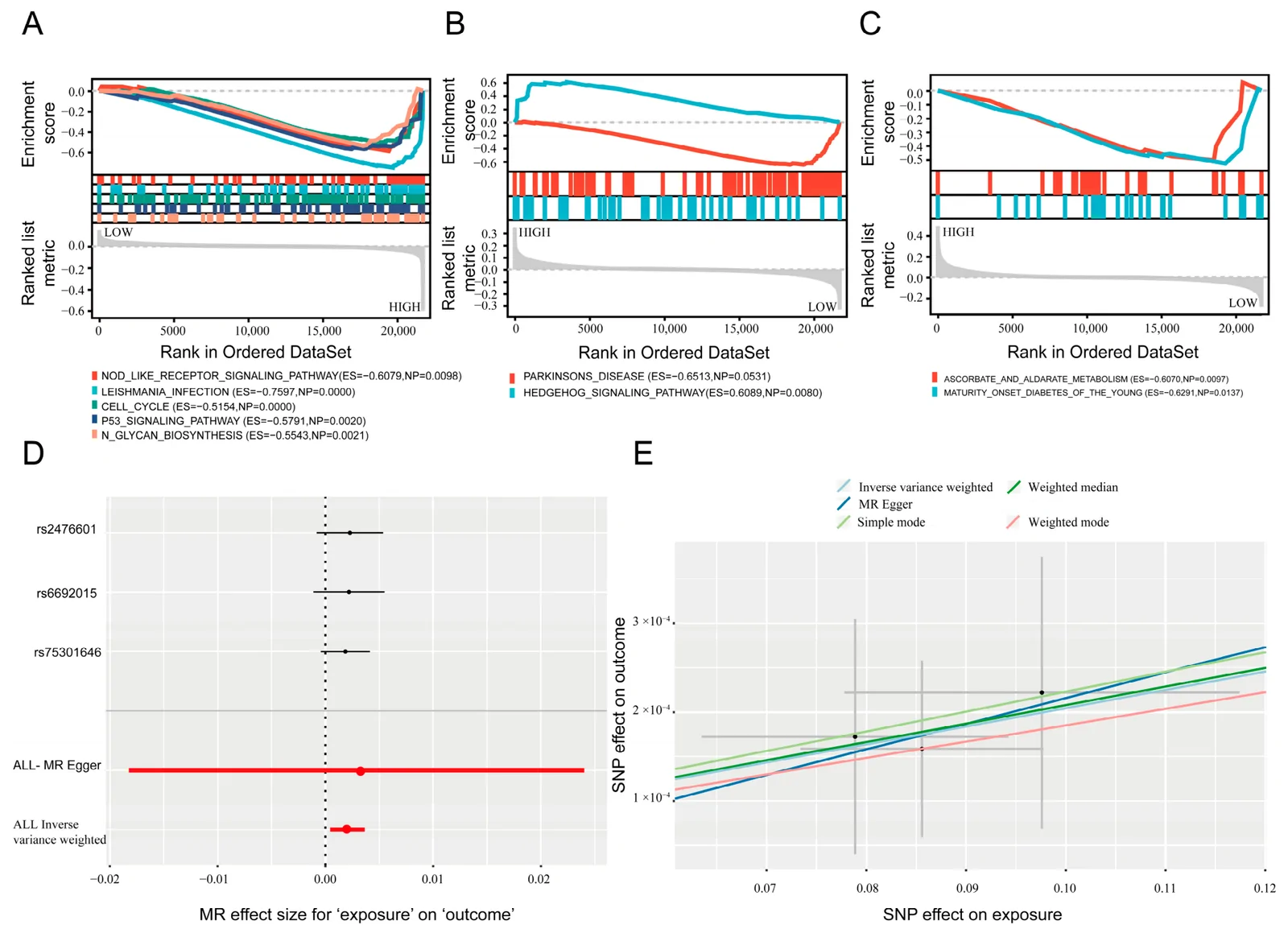
Pathway enrichment and causal associations of the core signature genes in AD. Gene set enrichment analysis (GSEA) profiling transcriptomic signatures associated with *RGS1* (**A**), *RNVU1-7* (**B**), and *VGF* (**C**). (**D**) Mendelian randomization forest plot assessing the causal effect of genetically predicted *RGS1* expression on AD risk. The black points and lines represent the MR effect estimates and 95% confidence intervals (CIs) for individual SNPs. The red points and lines represent the pooled overall MR effect estimates and 95% CIs combined across all SNPs using specific MR methods. (**E**) Mendelian randomization scatter plot illustrating the causal association between *RGS1* expression and AD risk across different MR methods.

GSEA was used to explore the architecture of consensus disease-associated candidate genes in the transcriptomic data of different *RGS1* expression levels. For *RGS1*, GSEA revealed significant negative enrichment in multiple immune and cellular stress pathways, indicating their upregulation in samples with elevated *RGS1* expression (Figure 4A). Mechanistically, this distribution pattern indicates the NOD-like receptor pathway and the Toll-like receptor pathway and the B cell receptor pathway belong to the innate and adaptive immune systems, respectively; that is to say, the NOD-like receptor signaling pathway (NES = −1.6588, FDR = 0.0476), and both the Toll-like receptor signaling pathway (NES = −1.5426, FDR = 0.0709) and the B cell receptor signaling pathway (NES = −1.5406, FDR = 0.0647) are transcriptionally linked to *RGS1* and active immune cells. Co-regulation of antigen processing and presentation (NES = −1.6234, FDR = 0.0604) with the complement and coagulation cascades (NES = −1.7615, FDR = 0.1197) shows that it is involved in immunological communication between the periphery and the center. In addition, *RGS1* was negatively correlated with apoptosis (NES = −1.5355, FDR = 0.0641) and the p53 signaling pathway (NES = −1.6698, FDR = 0.0584), so it may be related to stress-related neuronal frailty and the regulation of cell clearance. Additionally, there is a regulation of the actin cytoskeleton (NES = −1.3679, FDR = 0.187), and thus the modification of synaptic or dendritic structure by *RGS1* is probably a primary mechanism. Based on the results of GSEA, elevated *RGS1* expression is significantly associated with pathways involved in neuroinflammation, immune cell migration, and neuronal injury in AD.

In contrast, *RNVU1-7* expression did not yield any significantly enriched gene sets (all FDR > 0.25), suggesting a lack of robust pathway-level transcriptional coordination in this cohort (Figure 4B).

According to the GSEA, the expression of *VGF* is distributed relatively evenly, and it is relatively abundant in the areas of cranial antioxidant defense and neuroendocrine regulation, most notably ascorbate and aldarate metabolism (NES = −1.828, FDR = 0.0313). Additional terms included maturity-onset diabetes of the young (NES = −1.6237, FDR = 0.2025). Given that *VGF* is significantly downregulated in AD, these results demonstrate that reduced *VGF* expression correlates with the aberrant activation of specific metabolic pathways, including ascorbate and aldarate metabolism and maturity-onset diabetes of the young, hinting at a link between *VGF* depletion and metabolic dysfunction in AD (Figure 4C). Collectively, GSEA profiles highlight distinct pathway-level associations for *RGS1* and *VGF*, implicating immune modulation and metabolic/oxidative stress axes in AD pathogenesis. Full enrichment statistics are provided in Supplementary Table S5.

### 3.7. Mendelian Randomization Analysis of Consensus Genes and AD Susceptibility

To identify actionable therapeutic targets with clear functional roles in AD pathogenesis, we employed a step-wise evaluation strategy for the three core genes. Based on the prior GSEA results, *RNVU1-7* failed to yield significant or valid pathway enrichment and was thus excluded from further causal evaluation. Then, two-sample Mendelian randomization was used to determine whether the remaining candidates (*RGS1* and *VGF*) are the potential causal association with AD. No genome-wide significant single nucleotide polymorphisms (SNPs) meeting the conventional instrumental variable (IV) criteria (*p* < 5 × 10^−6^ and independent LD clumping) were found in the available cis-eQTL or AD GWAS repositories for *VGF*, and therefore it could not be used for MR analysis. Three different cis-eQTL regions for *RGS1* (rs2476601, rs669201,5 and rs75301646) have also been found and can be employed as genetic tools. According to the IVW analysis, it was found that an increased risk of AD is associated with more *RGS1* alleles (Figure 4D). A scatter plot has been added here to show the same trend in various MR methods, such as MR-Egger, weighted median, and weighted mode (Figure 4E). Although the confidence interval of the intercept for MR-Egger is relatively large due to having only three instruments (*n* = 3), there is consistency in the direction of the trend and no significant horizontal pleiotropy; generally speaking, the MR results have shown that increased expression of *RGS1* is associated with an increased risk of AD.

### 3.8. Cross-Cohort Validation and Classification Performance of RGS1

**Figure 5:**
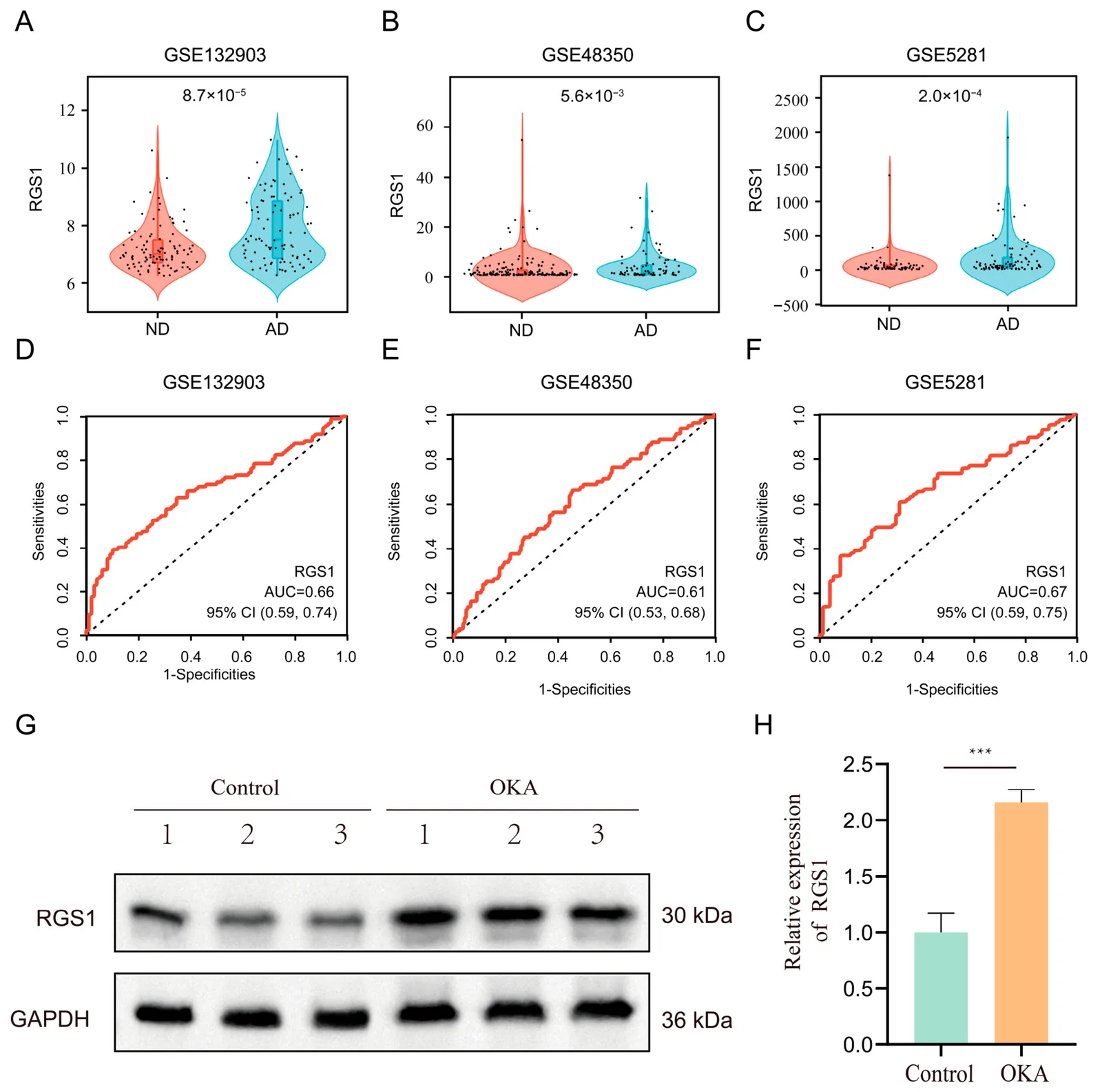
Transcriptional validation, predictive evaluation, and protein-level verification of *RGS1* in AD. Numerical evaluation of *RGS1* levels in the three specific genomic databases; statistical significance was determined using the Wilcoxon rank-sum test: GSE132903 (**A**), GSE48350 (**B**), and GSE5281 (**C**), the black dots represent individual sample data points. ROC curves were used to evaluate the discriminative ability of *RGS1* in GSE132903 (**D**), GSE48350 (**E**), and GSE5281 (**F**). (**G**) Western blot analysis comparing RGS1 protein expression between untreated control cells and OKA-induced AD model cells, with GAPDH serving as the loading control. (**H**) Densitometric quantification of RGS1 protein bands from three independent biological replicates, analyzed via ImageJ software (Version 1.53k). Statistical significance was determined using the one-way analysis of variance, and *** indicates *p* < 0.001.

To determine how much agreement there is in gene expression regulation at this level for *RGS1*, its expression in the initial cohort (GSE132903) and the two supplementary validation cohorts (GSE48350 and GSE5281) was analyzed. Based on the violin plot of *RGS1* abundance in AD specimens, it is shown that it increases gradually and reaches a level that is statistically different from neurologically normal controls in all three datasets (Figure 5A–C; all *p* < 0.05). Then, to determine the prediction power of *RGS1* for AD in the brain, an ROC curve was constructed. *RGS1* in the GSE132903 discovery series had an AUC of 0.66. The independent validation group in clinical practice has repeatedly confirmed this, and the AUC values are 0.61 for GSE48350 and 0.67 for GSE5281 (Figure 5D–F). Although single-gene *RGS1* demonstrates limited standalone discriminative ability, its reproducible upregulation and stable cross-cohort performance support its potential utility as a component within multi-gene discriminative panels for AD.

### 3.9. In Vitro Validation of RGS1 Upregulation in an OKA-Induced AD Cell Model

To validate the transcriptomic findings at the protein level, we established an in vitro AD model using SH-SY5Y neuroblastoma cells treated with okadaic acid (OKA). OKA is a particular PP2A phosphatase inhibitor that can cause tau hyperphosphorylation and other signs of neurodegeneration in AD. Western blot results show that the amount of RGS1 protein in the OKA group is higher than that in the normal control (NC) group (Figure 5G). Semi-quantitative densitometry shows that the level of RGS1 protein in the AD-mimicked state is 2.2 times that in NC (Figure 5H). The degree of protein elevation is in line with the expansion of gene expression that has been discovered and validated through both GEO arrays, providing support for the idea that RGS1 is overexpressed in cells under AD.

### 3.10. Immune Microenvironment Remodeling and Association with RGS1 Expression in AD

**Figure 6:**
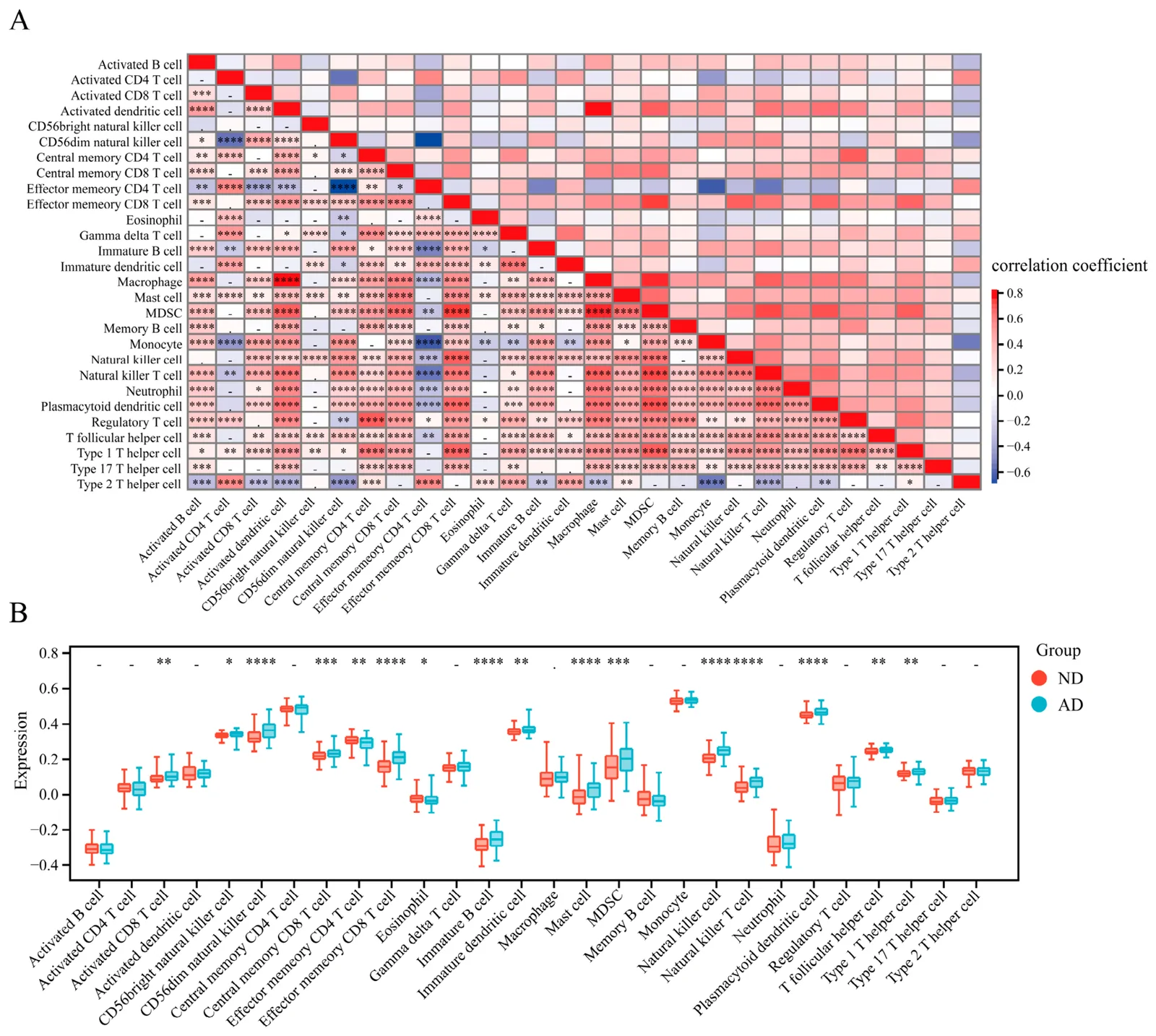
Characterization of the neuroimmune microenvironment in AD. (**A**) Heatmap showing the correlations among immune-cell signatures. Red indicates positive correlation, and blue indicates negative correlation. (**B**) Comparison of immune cell infiltration profiles between AD patients and ND reference groups. Statistical significance was determined using the Wilcoxon rank-sum test. Clinical significance of the overall transition is marked with asterisks (* *p* < 0.05, ** *p* < 0.01, *** *p* < 0.001, **** *p* < 0.0001). The symbol “-” indicates no change in quantity (*p* ≥ 0.05), and the mark “.” is for borderline significance (*p* < 0.1).

To investigate the immune mechanisms in AD, we performed transcriptome-based immune cell deconvolution using ssGSEA across the integrated cohorts. As shown in Figure 6A, the heatmap visualizes the correlations among different immune-cell signatures, revealing significant synergistic and antagonistic relationships between specific immune cell subpopulations in the CNS immune microenvironment due to AD. Next, we compared the immune-enrichment profiles between the AD and ND groups. As illustrated in Figure 6B, the relative abundance of several immune cells was significantly altered in AD samples compared to controls. Compared with ND subjects, the AD subject cohorts had an higher relative abundance of activated CD8^+^ T cell, CD56bright natural killer cell, CD56dim natural killer cell, central memory CD8^+^ T cell, effector memory CD8^+^ T cell, immature B cell, immature dendritic cell, mast cell, myelod-derived suppressor cells (MDSCs), conventional natural killer cell, natural killer T (NKT) cell, plasmacytoid dendritic cell, T follicular helper (Tfh) cell and Type 1 T helper cell. On the other hand, in the AD group, the number of effector memory CD4^+^ T cells was reduced to some extent, and then eosinophils dropped sharply (all *p* < 0.05). Most cohorts showed no clinical change in activated B cells, activated CD4^+^ T cells, activated dendritic cells, central memory CD4^+^ T cells, gamma delta T cells, macrophages, memory B cells, monocytes, neutrophils, regulatory T cells, type 17 T helper cells, and type 2 T helper cells. In conjunction with the above results, it can be seen that there is a pathogenic inflammatory microenvironment in AD, which shows an increase in antigen-presenting cells and memory T cells, as well as a decrease in the estimated abundance of regulatory cells and innate effector cells. The significant co-occurrence of elevated *RGS1* expression with specific neuroimmune enrichment profiles suggests that *RGS1* upregulation is closely coordinated with AD-associated immune microenvironment remodeling.

### 3.11. Multidimensional Correlation Analysis Links RGS1 Expression to Immune Microenvironment Remodeling

**Figure 7:**
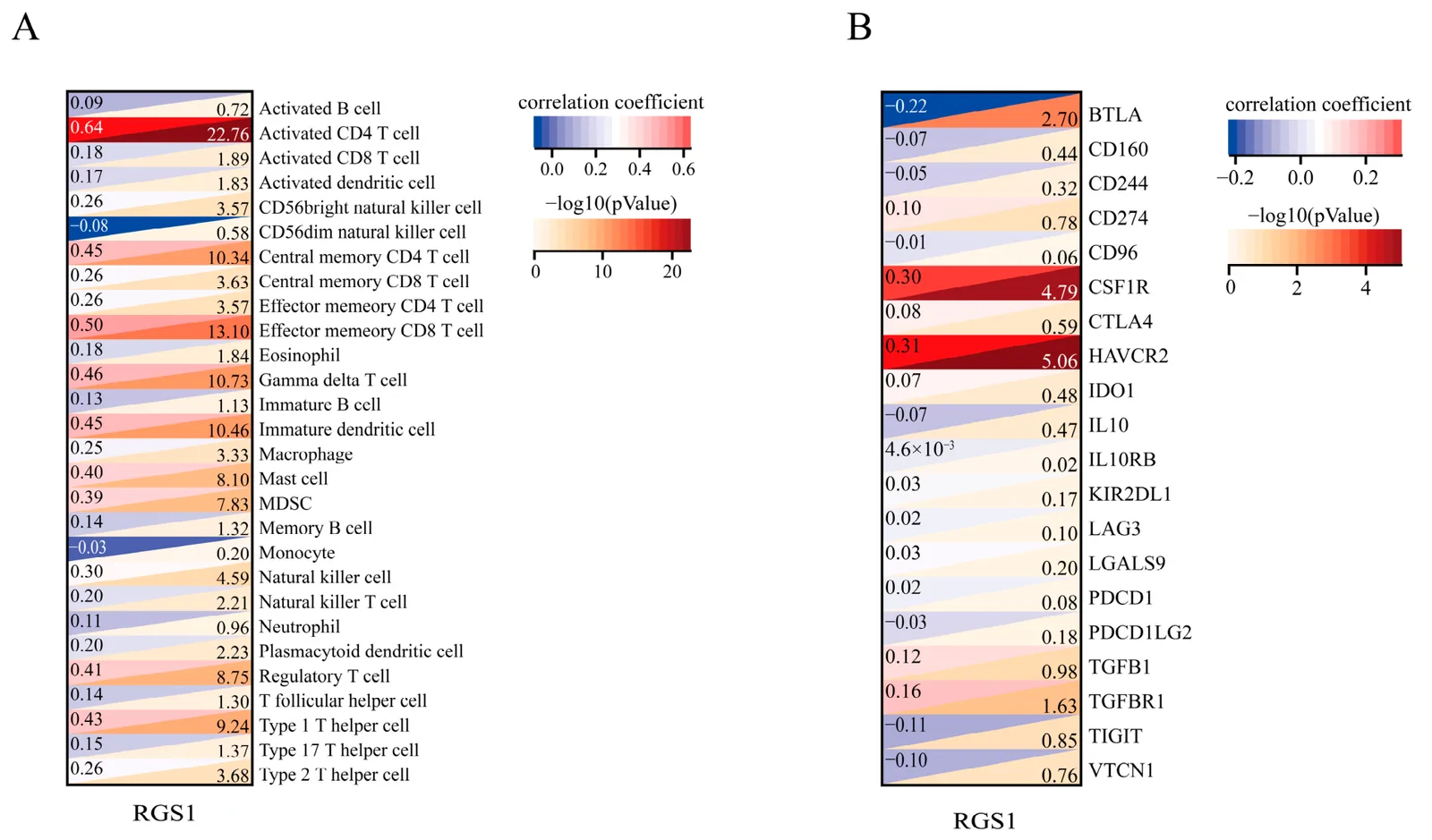

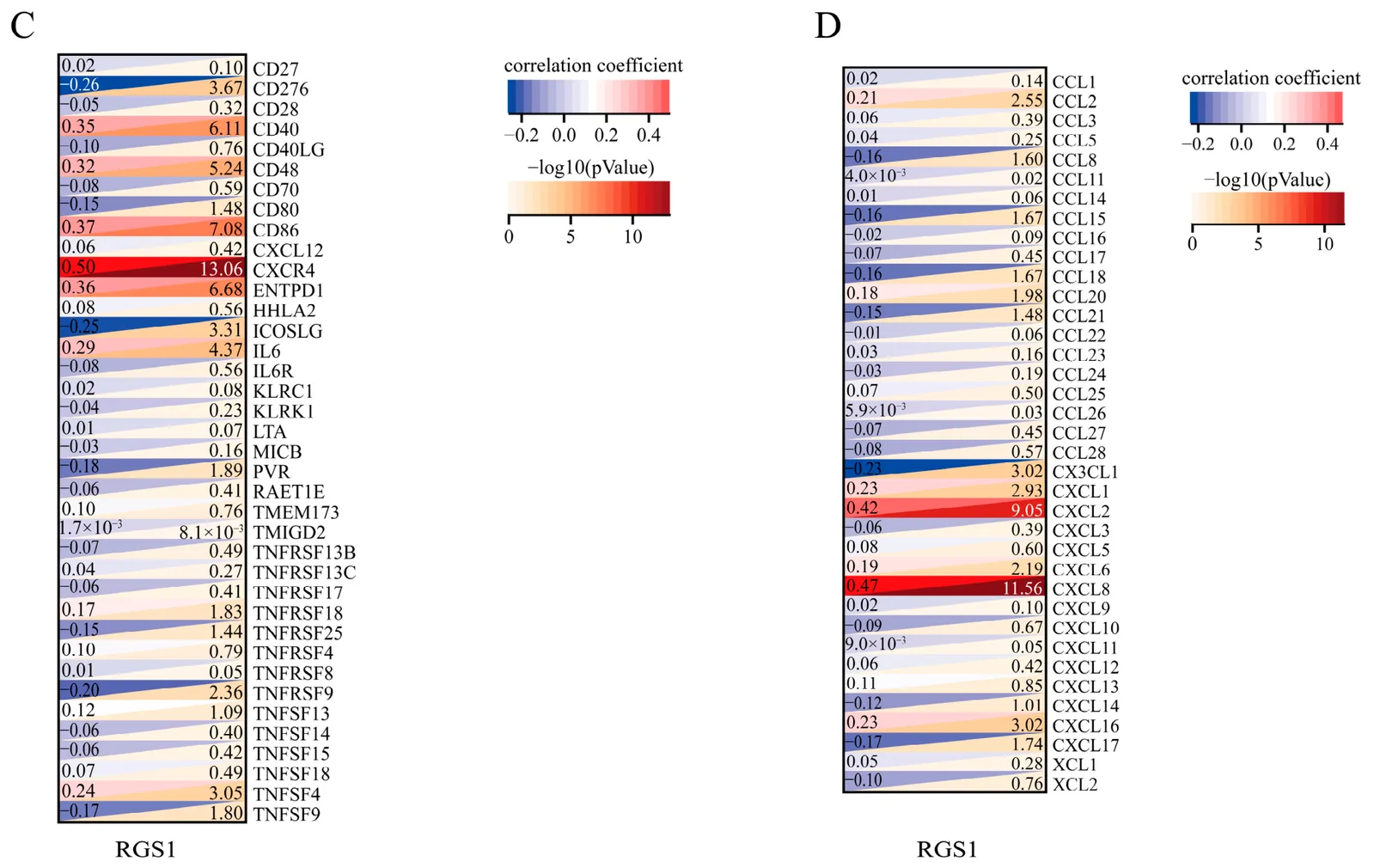
Correlation analysis between *RGS1* expression and immune-related markers. Scatter plots illustrating the correlations between *RGS1* and (**A**) specific immune cell types, (**B**) immunoinhibitory molecules, (**C**) immunostimulatory molecules, and (**D**) chemokines. Statistical significance of the correlation was determined by the Pearson correlation test. Quantitative correlation coefficients and corresponding *p*-values are displayed for each dataset.

To better understand the immunological context of *RGS1* in AD, a number of Spearman correlation analyses were performed to correlate the level of *RGS1* with all kinds of immune cell subsets and soluble mediators. There are 28 subsets of leukocytes, 20 suppressive cytokines, 38 stimulatory mediators, and 37 chemokines in the actual data set. *RGS1* is associated with 22 different cell subpopulations, and it particularly showed the strongest correlation with activated CD4^+^ T cell enrichment profiles (Figure 7A). Immunosuppression is associated with a large number of regulatory factors of *RGS1*, and among them, *HAVCR2* and *CSF1R* showed the strongest positive correlations (Figure 7B). In terms of pro-inflammatory markers, *RGS1* was statistically related to 14 different targets, and *CXCR4* had the highest degree of co-expression (Figure 7C). In the chemokine network, *RGS1* showed robust positive correlations with *CXCL8* and *CXCL2* (Figure 7D). These associations suggest a potential involvement of *RGS1* in AD-related neuroimmune crosstalk, warranting further mechanistic investigation.

## 4. Discussion

By using the large RNA-seq database, special statistical tools, and Mendelian randomization in this study, it was found that *RGS1* is a consistently upregulated transcript associated with AD susceptibility and neuroimmune microenvironment remodeling. Using the general method of multivariate logistic regression and LASSO-based feature selection, we identified a strong cluster of three indicators that are related to the core group of 302 dysregulated transcripts. Next, in order to obtain cross-sectional data on the abnormal expression pattern and genetic causality of AD susceptibility for *RGS1*, pathway enrichment analysis (GSEA) and reciprocal two-sample MR appraisal were used together. Complementary GSEA and immune deconvolution analyses further linked *RGS1* elevation to a coordinated pro-inflammatory and immunoregulatory transcriptional landscape, a pattern corroborated in an OKA-induced cellular model of AD-like pathology.

Logistic regression combined with a LASSO penalty is stable, and it has been used successfully to solve the problems of overfitting and multicollinearity in biomarker detection of vast RNA-seq data [21]. Although the first step is to screen for changes in gene expression in the disease alone, many are generalizations; they cannot be taken as statistical indicators of differential expression [22]. To address this, our dual-algorithm feature selection strategy effectively eliminated redundant information, yielding a reliable and cross-validated panel of biomarkers with high collective predictive accuracy. Secondly, compared with observational correlations, two-sample MR provides robust causal inference by minimizing confounding and reverse causation. Furthermore, we specifically utilized cis-eQTLs as instrumental variables because their direct biological effects on local gene expression significantly reduce the risk of horizontal pleiotropy [23,24]. Agreement was reached by IVW, MR-Egger, and the weighted median method; thus, our findings suggest that an increase in *RGS1* expression may not only be associated with neurodegenerative changes but also serve as a potential risk factor for AD. Therefore, prioritizing causal genes validated by human genetics is now widely recognized as a crucial strategy for the successful development of novel therapeutics for neurodegenerative diseases [25].

This causal framework is particularly timely and aligns with the latest methodological advancements in neurodegenerative research. Over the past two years, the field has increasingly recognized that traditional observational transcriptomics are heavily confounded by reverse causation and disease-related epiphenomena [26]. Consequently, recent high-impact studies have strongly advocated for the integration of multi-omics data with Mendelian randomization to prioritize genetically validated therapeutic targets for AD [27,28]. By adopting this cutting-edge integrative strategy, our study not only corroborates recent genomic consensus but also provides robust, non-confounded evidence that *RGS1* upregulation is a causal driver rather than a mere consequence of AD pathology.

From a functional perspective, our GSEA and immunoprofiling results align closely with the neuroimmune landscape previously described in [29]. High *RGS1* expression co-occurs with the NOD-like receptors, TLRs, B-cell receptors, the complement cascade, and p53-dependent apoptosis. Taken together, the above mechanisms are responsible for the initial recognition, antigen presentation, and neuroinflammatory responses following cellular injury [30,31]. ssGSEA shows that there is a significantly increased infiltration of activated CD8+ T cells, dendritic cells, and MDSCs in AD. Together, these immune cells orchestrate an antigen-driven inflammatory microenvironment [32]. Extensive immunological profiling has revealed that *RGS1* is positively correlated with activated CD4+ T cells, immune checkpoints (*HAVCR2*, *CSF1R*), and inflammatory chemokines (*CXCL8*, *CXCL2*). It has been shown that *RGS1* regulates GPCR-mediated leukocyte migration and tissue retention [33,34]. An increase in *RGS1* during AD progression may disrupt the normal Gα signal transduction pathway and therefore alter the migration of immune cells and promote an inflammasome cascade that is initiated by synaptic damage and tauopathies [35].

Furthermore, these immunoprofiling findings strongly resonate with the most recent paradigm shifts in AD research, which increasingly position peripheral immune cell infiltration and neuro-immune crosstalk at the core of AD pathogenesis [36,37]. Recent studies have extensively documented that the accumulation of exhausted or hyperactive T cells (such as CD4+ and CD8+ subsets) within the brain parenchyma exacerbates neuroinflammation and cognitive decline [38,39]. Notably, contemporary immunological research has clearly identified *RGS1* as a novel marker and promoting factor for CD8^+^ T cell exhaustion [40]. Viewed through the lens of these recently published papers, our observation that *RGS1* strongly correlates with active T cells and *HAVCR2* in AD provides a novel, genetically supported mechanism: *RGS1* may actively drive the exhaustion and aberrant tissue retention of infiltrating lymphocytes in the AD brain, thereby perpetuating a vicious cycle of neuroinflammation.

We have found that *RGS1* is elevated in all three clinical groups and in a cell line that is sensitive to OKA, but its classification performance (AUC values of 0.61-0.67) has been modest. The reasons for this are the complex genetic makeup of polygenic diseases, and a single candidate gene cannot capture the full heterogeneity of the disease [41,42]. Instead, *RGS1* likely functions merely as a single node within the highly complex neuroimmune regulatory network, rather than acting as a standalone diagnostic or prognostic biomarker [43]. Given its functional link to the chemokine signaling pathways and *CXCR4*, *RGS1* holds potential utility for patient stratification in emerging neuroimmune-targeted therapies. Furthermore, its clinical value could be maximized by integrating it into multi-modal diagnostic frameworks that combine molecular, histopathological, and neuroimaging data [44]. Importantly, our in vitro finding that RGS1 protein levels increased 2.2-fold under OKA-induced tau hyperphosphorylation strongly suggests its active functional involvement in AD-related cytoskeletal and metabolic stress [35].

Several limitations of this study should be acknowledged. First of all, conventional bulk RNA sequencing lacks the resolution to distinguish cell-type-specific gene expression. Therefore, in recent years, single-nucleus sequencing and spatial transcriptomics have been employed to identify the locations of *RGS1* transcripts in particular glial or neuronal cell populations [12,45]. Furthermore, while MR utilizing cis-eQTLs provides valuable unidirectional causal estimates, standard MR frameworks often lack sufficient statistical power to robustly detect and adjust for horizontal pleiotropy or linkage disequilibrium [9,46]. Our in vitro validation utilized an OKA-induced SH-SY5Y model to recapitulate tauopathy and metabolic stress, but this system does not fully capture amyloid-β dynamics, aging-related senescence, or the complex brain microenvironment [47]. Furthermore, although OKA is widely recognized for inducing AD-like pathology [48,49], our in vitro validation lacked concurrent measurement of classic AD markers such as hyperphosphorylated Tau. Consequently, we cannot entirely rule out the influence of non-specific cellular toxicity, highlighting the need for direct phenotypic validation in future studies to confirm that *RGS1* upregulation is specifically driven by the AD microenvironment. Finally, to definitively establish the causal relationship between *RGS1* expression, immune cell migration, and synaptic loss, future studies should employ targeted gene silencing or overexpression in primary microglia–neuron co-cultures and transgenic animal models [50].

In short, based on bioinformatics marker discovery, genetic causality analysis, and all-around immune characterization, it has been found that *RGS1* is an important gene regulating the immunological niche in AD. As shown in the results above, there is a molecular basis for the regulation of *RGS1*-mediated GPCR signaling in neuroinflammation and the suppression of disease development [35].

## Data Availability

Publicly available datasets were analyzed in this study. This data can be found in the Gene Expression Omnibus (GEO) database (https://www.ncbi.nlm.nih.gov/geo/, accessed on 18 November 2025) under the accession numbers: GSE132903, GSE48350 and GSE5281.

## Supplementary Materials

The following supporting information can be downloaded at https://www.mdpi.com/article/10.3390/cimb48080828/s1.
