# Supplementary material for: Integrative Transcriptomics and Mendelian Randomization Identify RGS1 as a Causal Immune Regulator in Alzheimer’s Disease
Source: Curr Issues Mol Biol. 2026 Aug 14;48(8):828. doi: 10.3390/cimb48080828 (PMC13511232; doi:10.3390/cimb48080828)
Supplement: Supplementary file 1 [file cimb-48-00828-s001.zip › Supplementary Table S1---Demographic and clinical characteristics in the three datasets.pdf]

Dataset ID：GSE132903

Sample：middle temporal gyrus (MTG; BA21)

Platform：GPL10558 Illumina HumanHT-12 V4.0 expression beadchip

Number of Subjects (AD / Control)：AD（n = 97）；Control（n = 98）

Number of Samples (AD / Control)：AD（n = 97）；Control（n = 98）

The Source of Samples (AD / Control):

	**ND Group (n = 74)**	**AD Group (n = 87)**
**medial temporal gyrus**	74 (100%)	87 (100%)

Demographic characteristic (AD / Control):

	**AD Group (n = 97)**	**ND Group (n = 98)**	***p***
**Age,y**			
mean	85.4	60.0	0.97
Range	70-98	70-102	
**Gender,n**			
Men	49 (50.5%)	50 (51.0%)	
Women	48 (49.5%)	48 (49.0%)	

Dataset ID：GSE48350

Sample： hippocampus, entorhinal cortex, superior frontal cortex, post-central gyrus

Platform：GPL570 [HG-U133_Plus_2] Affymetrix Human Genome U133 Plus 2.0 Array

Number of Subjects (AD / Control)：AD（n = 173）；Control（n = 80）

Number of Samples (AD / Control)：AD（n = 173）；Control（n = 80）

The Source of Samples (AD / Control):

	**ND Group (n = 173)**	**AD Group (n = 80)**
**Hippocampus**	43 (24.85%)	19 (23.75%)
**Entorhinal cortex**	39 (22.55%)	15 (18.75%)
**Superior frontal cortex**	48 (27.75%)	21 (26.25%)
**Post-central gyrus**	43 (24.85%)	25 (31.25%)

Demographic characteristic (AD / Control):

	**AD Group (n = 80)**	**ND Group (n = 173)**	***p***
**Age,y**			
mean	85.4	60.0	1.58E-26
Range	60-95	20-99	
**Gender,n**			
Men	33 (41.3%)	91 (52.6%)	
Women	47 (58.7%)	82 (47.4%)	

Dataset ID： GSE5281

Sample： entorhinal cortex, hippocampus, medial temporal gyrus, posterior cingulate, superior frontal gyrus, primary visual cortex

Platform： GPL570 [HG-U133_Plus_2] Affymetrix Human Genome U133 Plus 2.0 Array

Number of Subjects (AD / Control)： AD（n = 87）； Control（n = 74）

Number of Samples (AD / Control)： AD（n = 87）； Control（n = 74）

The Source of Samples (AD / Control):

	**ND Group (n = 74)**	**AD Group (n = 87)**
**Hippocampus**	13 (17.57%)	10 (11.49%)
**Entorhinal cortex**	13 (17.57%)	10 (11.49%)
**Superior frontal cortex**	11 (14.86%)	23 (26.44%)
**primary visual cortex**	12 (16.22%)	19 (21.84%)
**medial temporal gyrus**	12 (16.22%)	16 (18.39%)
**posterior cingulate**	13 (17.57%)	9 (10.34%)

Demographic characteristic (AD / Control):

	**AD Group (n = 87)**	**ND Group (n = 74)**	***p***
**Age,y**			
mean	79.8	78.4	0.40
Range	68-97	1-102	
**Gender,n**			
Men	50 (57.5%)	53 (71.6%)	
Women	37 (42.5%)	21 (28.4%)	
